# SVF-derived extracellular vesicles carry characteristic miRNAs in lipedema

**DOI:** 10.1038/s41598-020-64215-w

**Published:** 2020-04-29

**Authors:** Eleni Priglinger, Karin Strohmeier, Moritz Weigl, Carolin Lindner, Daniela Auer, Mario Gimona, Martin Barsch, Jaroslaw Jacak, Heinz Redl, Johannes Grillari, Matthias Sandhofer, Matthias Hackl, Susanne Wolbank

**Affiliations:** 1 Ludwig Boltzmann Institute for Experimental and Clinical Traumatology, AUVA Research Center, Linz/Vienna, Austria; 2 Austrian Cluster for Tissue Regeneration, Vienna, Austria; 3 TAmiRNA GmbH, Vienna, Austria; 4 School of Medical Engineering and Applied Social Science, University of Applied Sciences Upper Austria, Linz, Austria; 5 Spinal Cord Injury and Tissue Regeneration Center Salzburg (SCI-TReCS), Salzburg, Austria; 6 GMP Laboratory, Paracelsus Medical University, Salzburg, Austria; 7 Austrian Center for Lipedema, Linz/Vienna, Austria; 8 Institute of Molecular Biotechnology, Department of Biotechnology, BOKU - University of Natural Resources and Life Sciences, Vienna, Austria

**Keywords:** Mechanisms of disease, miRNAs, Biomarkers, Diseases, Biomarkers

## Abstract

Lipedema is a chronic, progressive disease of adipose tissue with lack of consistent diagnostic criteria. The aim of this study was a thorough comparative characterization of extracellular microRNAs (miRNAs) from the stromal vascular fraction (SVF) of healthy and lipedema adipose tissue. For this, we analyzed 187 extracellular miRNAs in concentrated conditioned medium (cCM) and specifically in small extracellular vesicles (sEVs) enriched thereof by size exclusion chromatography. No significant difference in median particle size and concentration was observed between sEV fractions in healthy and lipedema. We found the majority of miRNAs located predominantly in cCM compared to sEV enriched fraction. Surprisingly, hierarchical clustering of the most variant miRNAs showed that only sEVmiRNA profiles – but not cCMmiRNAs – were impacted by lipedema. Seven sEVmiRNAs (miR–16-5p, miR-29a-3p, miR-24-3p, miR-454-p, miR–144-5p, miR-130a-3p, let-7c-5p) were differently regulated in lipedema and healthy individuals, whereas only one cCMmiRNA (miR-188-5p) was significantly downregulated in lipedema. Comparing SVF from healthy and lipedema patients, we identified sEVs as the lipedema relevant miRNA fraction. This study contributes to identify the potential role of SVF secreted miRNAs in lipedema.

## Introduction

Lipedema is a chronic, progressive disease characterized by bilateral, symmetrical, disproportional deposition of adipose tissue in the extremities and buttocks^1^. Patients suffer from pain, reduced joint mobility, hematoma, edema and psychological impacts^2^. It was first described in 1940 as a connective tissue disorder, characterized by fluid being collected in the interstitium instead of entering into lymphatics^3^. This excess fluid in the interstitium potentially leads to growth of adipose tissue and hypoxia, which in turn might enhance angiogenesis of pathologic vessels^4,5^. The area of lymphatic vessels and the number of blood vessels were found increased in non-obese lipedema patients compared to controls^6^. Examination of adipose tissue from lipedema patients demonstrated hypertrophic adipocytes, crown-like structures and increased number of macrophages^6–8^.

Besides functioning as an energy storage, white adipose tissue (WAT) responds differentially to physiological and pathological metabolic changes by secreting a large diversity of proteins, hormones, lipids, non-coding ribonucleic acids (RNAs) – including microRNAs (miRNAs) – and extracellular vesicles (EVs)^9,10^. Small EVs (sEVs) are a fraction of 70–150 nm sized, membrane-enclosed particles, which contain cell-type specific proteins, enzymes, growth factors, cytokines, lipids, as well as coding and non-coding RNAs. It has been repeatedly reported, that WAT-derived vesicular miRNAs are involved in metabolic regulations^11,12^ and adipose tissue is considered a significant source of circulating sEV-miRNAs^11^. By acting in an autocrine, paracrine as well as systemic manner, these factors can contribute to metabolic abnormalities, modulation of osteogenic differentiation, inhibition of adipogenesis, adipocyte hypertrophy and infiltration of immune cells^13–15^. Many molecules released by adipose tissue originate from non-adipocyte cells, such as endothelial and immune cells present in the stromal vascular fraction (SVF) of adipose tissue^9,13^.

Critical issues are the unknown etiology of the disease and the lack of consistent diagnostic criteria leading to misdiagnosis in many cases. Clinicians must consider multiple criteria during the physical examination and conducting the medical history. A frequent comorbidity with 21.5% is cardiac disease^16^, however the risk of diabetes, dyslipidemia and hypertension is low despite an obese median body mass index (BMI)^1,17^.

To our knowledge, there are no blood-based parameters to identify lipedema patients so far. Due to this lack of systemic differences, we previously focused on the diseased tissue itself, specifically on the cellular components in which the disease is manifested and analyzed SVF cells isolated from liposuction material of lipedema patients. The isolated cells showed a reduced adipogenic differentiation potential as compared to healthy controls, similar to Bauer *et al*.^18^. However, we observed a higher cell yield after isolation, which might be due to the enhanced cell number of cluster of differentiation (CD) 90 (mesenchymal) and CD146 (endothelial) positive cells^7^. Moreover, the proliferation capacity of adipose-derived stem/progenitor/stromal cells (Ki67 + CD34 + cells) was enhanced in lipedema tissue^8^.

miRNAs are small non-coding RNAs that regulate gene expression through RNA interference. More than 70% of all human coding genes are under the control of miRNAs. Intracellular miRNA transcription and subsequent miRNA release from cells within EVs and protein complexes, which protect the RNA cargo from degradation, can inform about the onset and progression of human diseases^19^. Several studies have shown that either the overall number of EVs and/or the relative miRNA cargo can be altered in response to stress such as senescence or subtoxic liver damage^20,21^. Nevertheless, several studies suggest that the majority of extracellular miRNAs are contained in protein complexes rather than exosomes/microvesicles^22,23^.

Based on our previous knowledge about the relevance of SVF cells in lipedema and the potential link between extracellular miRNA profiles and disease phenotypes, the aim of this study was to perform a thorough characterization of the extracellular miRNA fraction produced by the SVF in healthy (control) individuals and lipedema patients. For this, we analyzed 187 extracellular miRNAs in the concentrated conditioned medium (cCM) and specifically in sEVs isolated thereof by size exclusion chromatography (SEC), to determine the relevant fraction containing the lipedema discriminating miRNAs.

## Methods

### SVF isolation

Table 1 provides patient characteristics. Subcutaneous adipose tissue was obtained during routine outpatient liposuction procedures from the hips and outer thighs (“saddlebags”) under local tumescence anaesthesia. Tumescence solution (per liter) contained 3.3 mg Volon-A (Dermapharm), 1 vial Suprarenin 1 mg/mL (Sanofi-Aventis), 15 mL bicarbonate 8.4% (Fresenius Kabi) and 23.3 mL Xylocaine 1% (GebroPharma). The harvesting cannulas were triport and 4 mm in diameter (MicroAire System power-assisted liposuction). 100 mL liposuction material was transferred to a blood bag (Macopharma, Langen, Germany) and washed with an equal volume of phosphate buffered saline (PBS) to remove blood and tumescence solution. Next, for tissue digestion PBS was replaced with 0.2 U/mL collagenase NB4 (Nordmark, Uetersen, Germany) dissolved in 100 mL PBS containing Ca^2+^/Mg^2+^and 25 mM N-(2-hydroxyethyl) piperazine-N′-(2-ethanesulfonic acid) (HEPES; Sigma, Vienna, Austria), resulting in a final collagenase concentration of 0.1 U/mL. The blood bag was incubated at 37 °C under moderate shaking (180 rpm) for 1 h. The digested tissue was transferred into 50 mL-tubes (Greiner, Kremsmünster, Austria). After centrifugation at 1200 × *g* for 7 min the supernatant was removed and the cell pellet was incubated with 100 mL erythrocyte lysis buffer for 5 min at 37 °C to eliminate red blood cells. The supernatant after centrifugation for 5 min at 500 × *g* was aspirated and the cell pellet was washed with PBS and filtrated through a 100-µm cell strainer (Greiner). After another centrifugation step at 500 × *g* for 5 min the supernatant was removed. The isolated SVF was resuspended in medium filtrated (0.22 µm; Merck, Vienna, Austria) before: DMEM-low glucose (Lonza, Vienna, Austria) containing 10% fetal calf serum (FCS; Sigma, Vienna, Austria), 2 mM L-glutamine (Lonza). Cell number was determined using trypan blue exclusion and quantification in a cell counter (TC-20, Bio-Rad, Vienna, Austria).

**Table 1:** Characteristics of control and lipedema patients.

Characteristics	control	lipedema
N	3	3
Sex (M/F)	female	female
Age (years)	35.0 ± 18.4	43 ± 14.2
BMI (kg/m²)	24.2 ± 5.0	28.0 ± 4.5
Type I-IV	—	3
Stage 1–2	—	3

The cohort underwent liposuction in this study were characterized regarding sex, age, and BMI and for lipedema patients the type and stage of lipedema. Data are presented as mean ± SD.

### Collection of cell supernatants

For miRNA analysis, DMEM-low glucose containing 10% FCS, 2 mM L-glutamine was filtrated using 0.22 µm filter cups. Preliminary experiments usingphotometric analysis of total RNA concentration (NanoDrop, Thermo Fisher Scientific, Vienna, Austria) and subsequent analysis with the HS RNA Kit (Agilent) showed a negligible RNA concentration of medium not conditioned by cells, in comparison to cell conditioned medium (Supplementary Fig. S1), a contribution of FCS-derived miRNAs was considered unlikely. Therefore, we decided to include FCS in our medium to provide best possible conditions for SVF culture. 6 × 10^6^ freshly isolated SVF cells were seeded in 22 mL filtrated medium in a T175 flask. The conditioned medium (CM) was collected after 24 h in a 50 mL-tube and centrifuged at 500 × *g* for 15 min (Eppendorf, 5804 R; Vienna, Austria) to remove cellular debris. After transfer to a new tube (VWR, Vienna, Austria) and centrifugation at 14000 × *g* for 15 min at 4 °C (HeraeusMultifuge X3R; Thermo Fisher Scientific) the supernatant was filtered through a 0.8 µm strainer (VWR) and immediately frozen and stored at −80 °C until further analysis.

### Processing of supernatants for miRNA analysis

CM was concentrated to 1 mL using centrifugation-based ultrafiltration, resulting incCM. Briefly, 20 mL of CM were transferred to an Amicon 30 kDa ultrafiltration column (Merck, Darmstadt, Germany) and centrifuged at room temperature for 15 minutes. Residual volumes were measured and diluted to 1 mL using sterile PBS solution. sEVs were subsequently enriched from cCM by SEC using qEV70s single columns (Izon Science, Christchurch, New Zealand): 150 µL of cCM were loaded based on recommendations from the manufacturer, and fraction 8–11 were collected and pooled to obtain the fraction of enriched sEVs. Nanoparticle tracking analysis (NTA) was performed on all sEV samples to determine particle size and concentration.

### Nanoparticle tracking analysis (NTA)

For determination of size and concentration of particles CM, cCM and sEV fractions obtained by SEC were analyzed onZetaView PMX 110 V3.0 particle analyseras previously described^24^ (Particle Metrix GmbH, Meerbusch, Germany).

### MACSPlex surface protein profiling

The MACSPlexExosome Kit (MiltenyiBiotec, BergischGladbach, Germany) is a bead-based multiplexed FACS-based assay for the analysis of surface markers present on EVs. We have used the MACSPlex kit according to the manufacturer’s instruction and following a validated standard operating procedure with 5 × 10^7^ to 5 × 10^8^ particles as input. Data acquisition was conducted on a FACS Canto II (BD Biosciences). Data normalization was directed towards to CD9/CD63/CD81 to assess the level of specific marker positive EVs. Isotype control normalization was performed essentially as described^25^.

### Total RNA extraction

Total RNA extraction was performed using 200 µl cCM or 200 µL pooled sEV fractions (fraction 8–11) together with the miRNeasy mini kit (Qiagen, Hilden, Germany). Synthetic oligonucleotides obtained from the miRCURY Spike-In kit (Qiagen) were added to the Qiazollysis buffer (Qiagen) before homogenization of the cCM/sEV samples. Glycogen (5 mg/mL) was added to the chloroform extract at 1:100 dilution to enhance precipitation. All other steps were performed according to the recommendations of the manufacturer. Total RNA was eluted in 30 µL nuclease-free water and stored at −80 °C in low-bind tubes (Eppendorf, Hamburg, Germany) until further analysis.

### MiRNA analysis

Reverse transcription (RT) of total RNA to complementary desoxyribonucleic acid (cDNA) was performed in 50 µL reaction volumes using the miRCURY RT kit (Qiagen) and 10 µL total RNA as input. The RT mix was incubated at 42 °C for 60 min. The resulting cDNA was stored at −20 °C until further analysis. Real-time quantitative PCR (qPCR) reactions were prepared using the miRCURY SYBR Green mastermix and a final cDNA dilution of 1:50. PCR amplification was performed in 384-well pick&mix plates (Qiagen) with customized selection of 192 LNA-enhance primers to detect 187 endogenous miRNAs and 5 spike-in controls per sample. The mastermix/cDNA sample was added to 384-well plates using an epMotion liquid handling robot (Eppendorf). Following the preparation of plates, an incubation at 4 °C for at least 60 min was performed before the plates were analyzed on a LightCycler 480 II (Roche, Basel, Switzerland) using 45 cycles and a temperature profile recommended by the manufacturer for miRCURY SYBR Green mastermix. Cycle quantification (Cq)-values were called using the second-derivate maximum method. Data quality was assessed using spike-in controls (Supplementary Fig. S2) to determine RNA extraction efficiency, enzymatic inhibition, and overall variability. Data normalization was performed using the global mean (i.e. average Cq-value for all endogenous miRNAs with Cq < 35) to obtain delta Cq-values. In preliminary tests, three technical replicates of cell conditioned medium from one donor were analyzed for four different miRNAs. High reproducibility between technical replicates of the same donor was observed (Supplementary Fig. S3), wherefore we analyzed one replicate of the isolated SVF of each donor for further analyses.

### MiRNA target analysis

MicroRNA target analysis was performed using the publicly available tool miRNet^26^. The list of differentially regulated miRNAs was uploaded as miRbase IDs and analyzed for gene targets with experimental verification. No tissue was selected as “context”. The node degree filter was set to 2.0 and gene enrichment was performed using hypergeometric testing and the reactome pathway database/gene classification^27^. Gene-pathway enrichment was also tested using the empirical sampling method to lower the risk of false-positives.

### Statistical analysis

Exploratory data analysis was performed using ClustVis^28^. Global-mean normalized delta Cq-values (dCqs) were used for clustering analysis based on Euclidean distance and complete linkage. Unit variance row scaling was applied. Statistical analysis was performed using unpaired two-sided t-tests.

### Ethic statement

All experimental protocols concerning human adipose tissues were approved by the Ethics Committee of the Province of Upper Austria (application/approval number 200, 12.05.2005 and 19.05.2014). Informed consent was obtained from all patients, if subjects were under 18, from a parent and/or legal guardian. All methods involving humans in this study were performed in accordance with relevant guidelines and regulations.

## Results

### Clinical characteristics and sample collection

**Figure 1:**
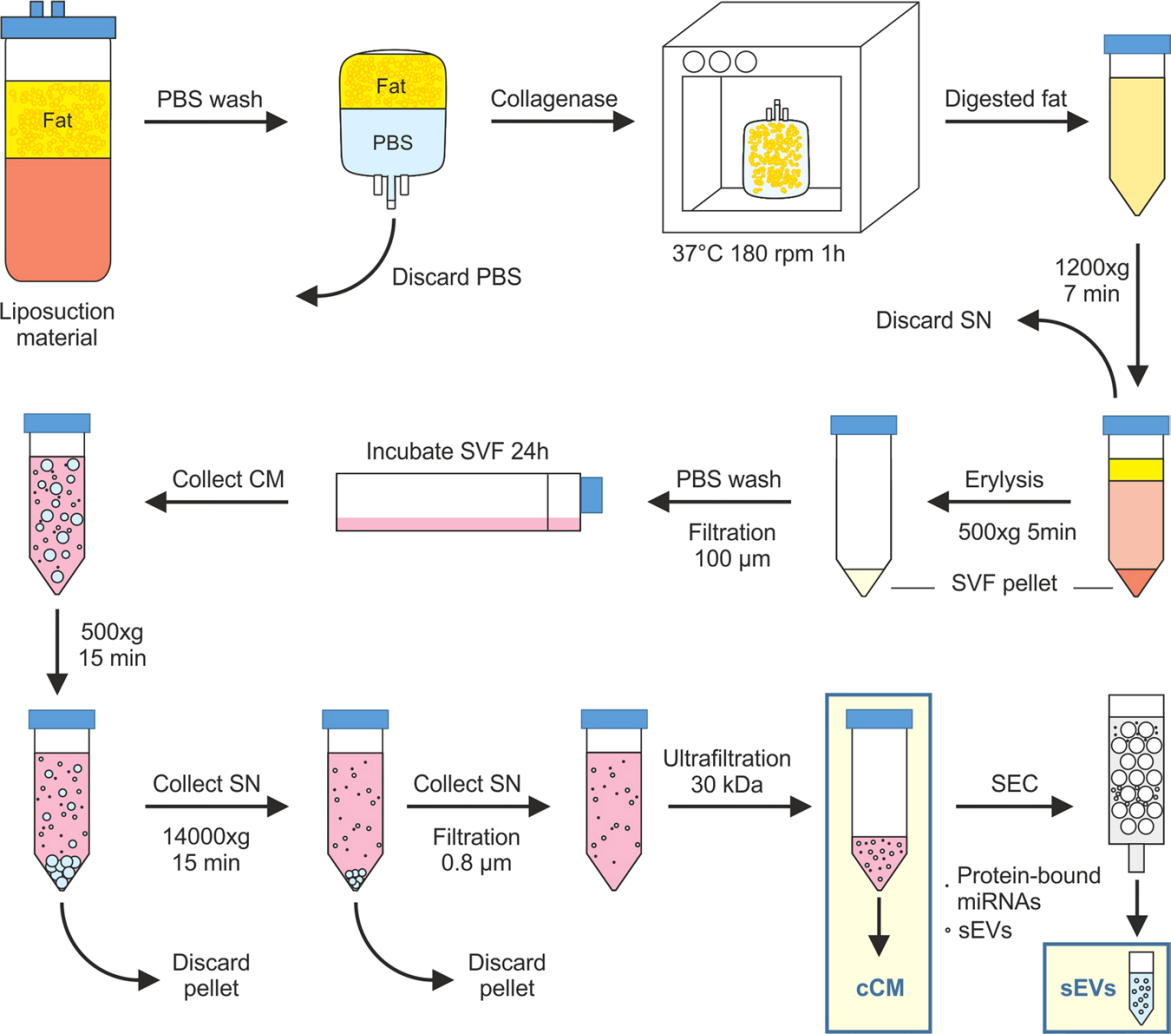
Experimental setup for SVF isolation, collection of cell supernatants and processing of supernatants for miRNA analysis.Subcutaneous adipose tissue was obtained during routine outpatient liposuction procedures. The liposuction material was washed and digested with collagenase. After centrifugation of the digested fat, the cell pellet was incubated with erythrocyte lysis buffer. After another centrifugation step, the cell pellet was washed and filtered and the isolated stromal vascular fraction (SVF) was incubated for 24 h in sterile filtered medium. Conditioned medium (CM) was collected and after two centrifugation steps and filtration, it was concentrated using centrifugation-based ultrafiltration to obtain concentrated CM (cCM). Small extracellular vesicles (sEVs) were enriched from cCM by size exclusion chromatography (SEC).

The cohorts that underwent liposuction in this study were all female, 3 healthy individuals (=control) and 3 lipedema (=lipedema) patients. Average age was 35.0 ± 18.4 for the control and 43 ± 14.2 years for the lipedema group. The BMI of the control group was 24.2 ± 5.0 kg/m^2^ and 28.0 ± 4.5 kg/m^2^ for the lipedema group. The BMI is similar in lipedema and healthy individuals (p-value 0.39), additionally the standard deviation was similar in healthy and lipedema indicating a comparable distribution within the cohorts. The slight enhancement in the lipedema cohort can be explained by the excessive fat accumulation in the thighs due to the disease. The three lipedema patients were categorized as type I-IV and stage 1–2 (according to Dr. Barsch and Dr. Sandhofer; Table 1). For collection of the samples (cCM and sEVs) all steps from liposuction (site, anesthesia, cannula) to SVF isolation, cultivation and preparation were performed consistently for each sample (Fig. 1).

### sEVs do not change in size or number in lipedema patients

In order to determine the relevance of SVF miRNAs in lipedema, we collected CM of adipose tissue derived SVF cells. NTA was employed to analyze particle size and concentration. By ultrafiltration a 17-fold enrichment of particles was achieved in the hereinafter called cCM (Supplementary Fig. S4a). sEVs were successfully separated from proteins of the cCM by SEC (Supplementary Fig. S4b). Fractions 8–11 showed the highest particle count and were pooled for further analysis. The particle diameter did not differ between cCM and sEVs (Supplementary Fig. S4c,d), indicating a comprehensive sEV enrichment. Particle size obtained by SEC ranged between 50 nm and 700 nm. The median particle size was determined to be 165 nm (+/−2.85) for the control group and 167 nm (+/− 10.78) for the lipedema group (Table 2). Similarly, no significant difference in particle concentration was observed between both groups, despite a trend towards higher concentrations in the lipedema group (FC = 1.41). Surface marker analysis of the EV preparation using a multiplex bead-based flow cytometry assay revealed also no significant differences between both groups (Supplementary Fig. S5).

**Table 2:** Particle number and size of purified extracellular vesicles (EVs) from control and lipedema analyzed by nanoparticle tracking analysis.

Group		Particle number [Particles/ul]	Median particle size [nm]	Mean particle size [nm]
**control 1**		1.63E + 14	161	182
**control 2**		2.87E + 14	166	186
**control 3**		2.73E + 14	168	191
**lipedema 1**		5.07E + 14	153	167
**lipedema 2**		2.97E + 14	179	197
**lipedema 3**		2.20E + 09	168	187
**control vs. lipedema**	**Effect Size (fold change)**	1.11	1.01	0.99
**control vs. lipedema**	**p-value (2-sided t test)**	0.87	0.85	0.81

Nanoparticle tracking analysis was applied to the small EVs (sEVs) samples from controls and lipedema patients to determine concentration and size of purified nanoparticles. No significant differences were observed in particle concentration and particle size between controls and lipedema patients.

### The majority of miRNAs are more abundant in cCM compared to sEV fraction

**Figure 2:**
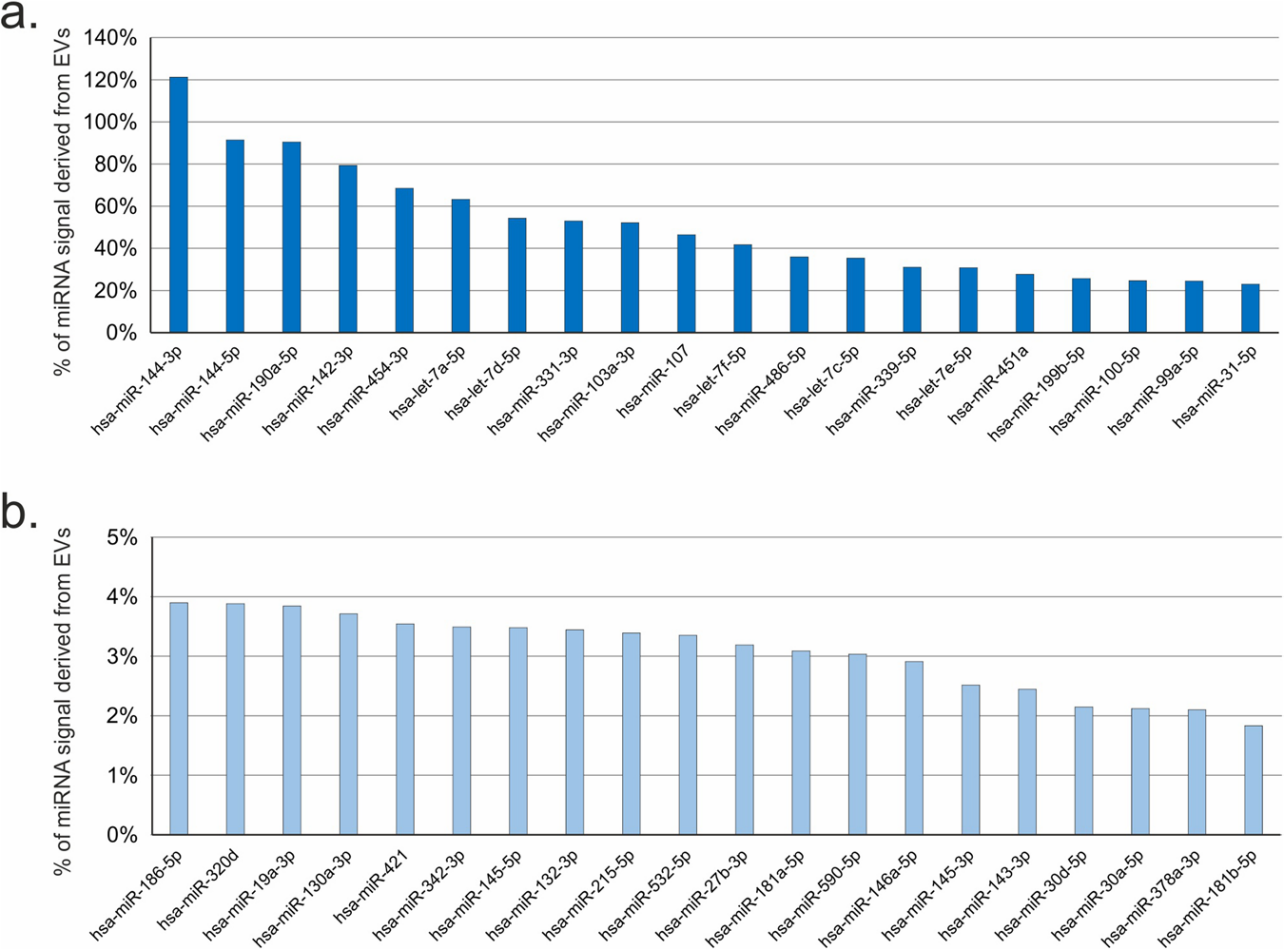
Analysis of microRNA (miRNA) signal origin. miRNAs signals obtained in small extracellular vesicles (sEVs) were compared against the total miRNA signal in concentrated conditioned medium (cCM) and expressed as %. (**a**) Top 20 miRNAs with highest EV-derived signal: Three miRNAs, for which more than 90% of the signal in cCM originated from the sEV enriched fraction (miR-144-3p, miR-144-5p, miR-190a-5p), and 6 further miRNAs where more than 50% of the signal was derived from the sEV fraction could be found. (**b**) Bottom 20 miRNAs with signals mostly derived from non-EV miRNAs: 83 out of 133 miRNAs were highly enriched in the non-sEV fraction as less than 10% of the total signal was obtained from the sEV fraction.The 20 miRNAs with the lowest signal, were below 4%.

192-plex RT-qPCR panels were used to quantify 187 miRNAs and 5 control assays in all 12 samples (6x cCM and 6x sEV). Spike-in controls showed homogenous values across all 12 samples with low variability, demonstrating low analytical variability, no enzymatic inhibition and overall high quality of the analysis (Supplementary Fig. S2). In the cCM samples 182 out of 187 miRNAswere above the limit of detection, while 133 miRNAs were detectable in the sEV fractions. These 133 miRNAs were also detectable in the cCM. Therefore, this set of 133 miRNAs was used for all further statistical analyses. The global mean normalized miRNA levels in cCM samples were interpreted as the total miRNA signal, since the analysis captures miRNA cargo in EVs as well as protein complexes. In order to determine how much of the total signal came from the sEV fraction, the sEVenriched miRNA signals were related to the cCMmiRNA signals and expressed as percentage of miRNA signal derived from sEV. Figure 2 shows the top and bottom 20miRNAs (Fig. 2a,b) in sEV and cCM. Supplementary Table 1 presents the results for all 133 miRNAs. We identified three miRNAs, for which more than 90% of the signal in cCM originated from the sEV enriched fraction (miR-144-3p, miR-144-5p, miR-190a-5p), and 6 further miRNAs where >50% of the signal was derived from the sEV fraction. However, 83 out of 133 miRNAs were highly enriched in the non-sEV fraction as <10% of the total signal was obtained from the sEV fraction. This set of miRNAs included for example the miR-30 family and miR-23-24-27 family of miRNAs.

### Clustering and differential expression analysis based on cCM and sEVmiRNA profiles

**Figure 3:**
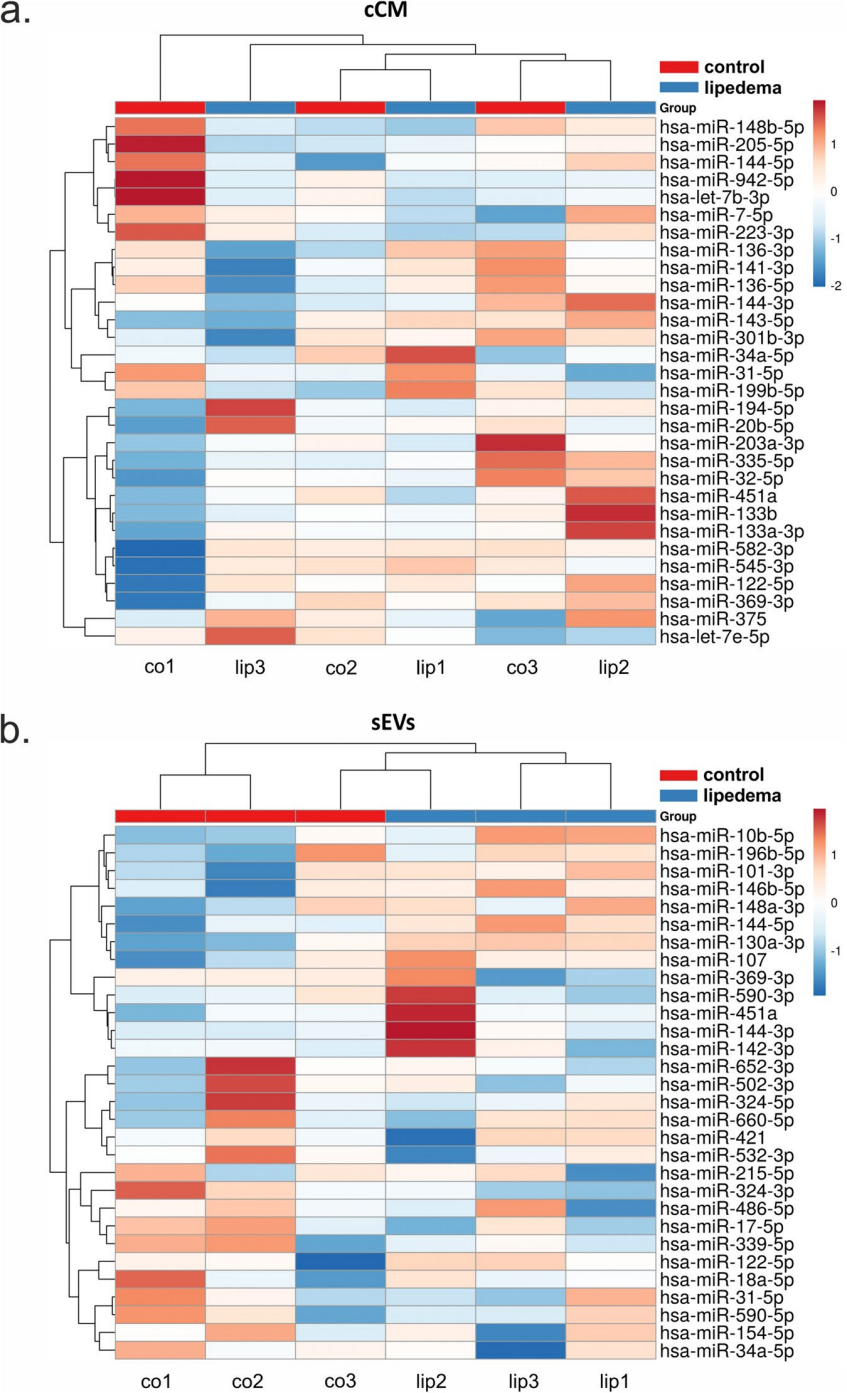
Hierarchical clustering of samples based on microRNA (miRNA) profiles observed in concentrated conditioned medium (cCM) or small extracellular vesicles (sEVs) from control (co) and lipedema (lip). The 30 most variant (according to coefficient of variation) miRNAs in cCM (**a**) and sEVs (**b**) were used for clustering analysis (Pearson correlation, average linkage). Rows are centered and unit variance scaling is applied to the global mean normalized expression values. Color indicates relative up- (red) or down-regulation for each miRNA (row). There were no clusters corresponding to the groups in the cCM miRNA data (**a**), while in the sEV enriched miRNA data 2 control samples clustered and 2 lipedema samples clustered (**b**).

**Figure 4:**
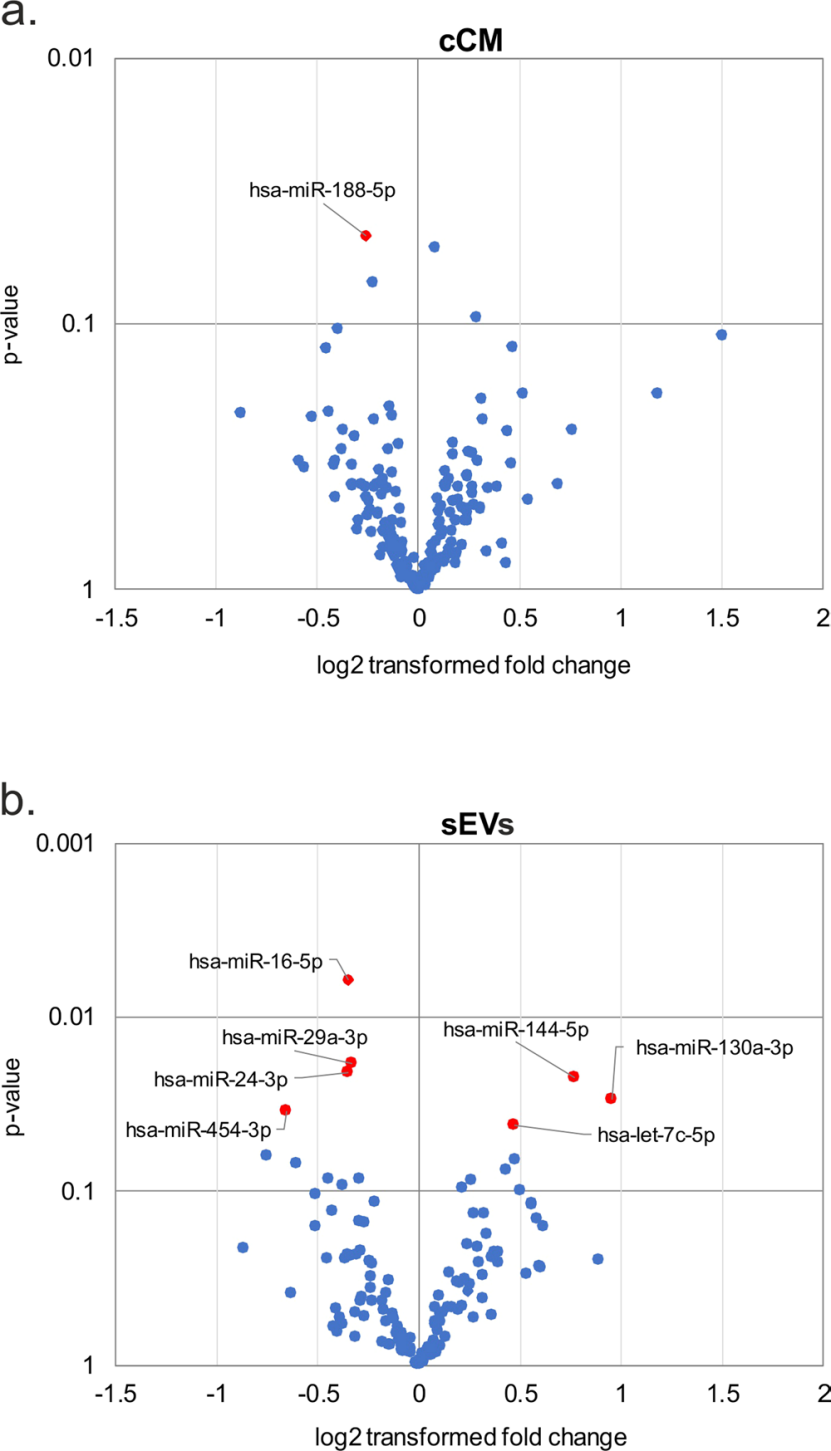
Differential secretion of extracellular microRNAs (miRNAs) between control and lipedema. 182 miRNAs were analyzed by RT-qPCR in concentrated conditioned medium (cCM) (**a**) and small extracellular vesicles (sEVs) (**b**) fractions. Volcano plots depict the log2 transformed fold change and p-value for the measured miRNAs. The overall trend in up- and down-regulated miRNAs between lipedema and control was balanced for both the cCM (**a**) and sEV (**b**) fraction. P-value < 0.05 are highlighted in red. n = 3 per group, unpaired two-tailed t-test.

**Figure 5:**
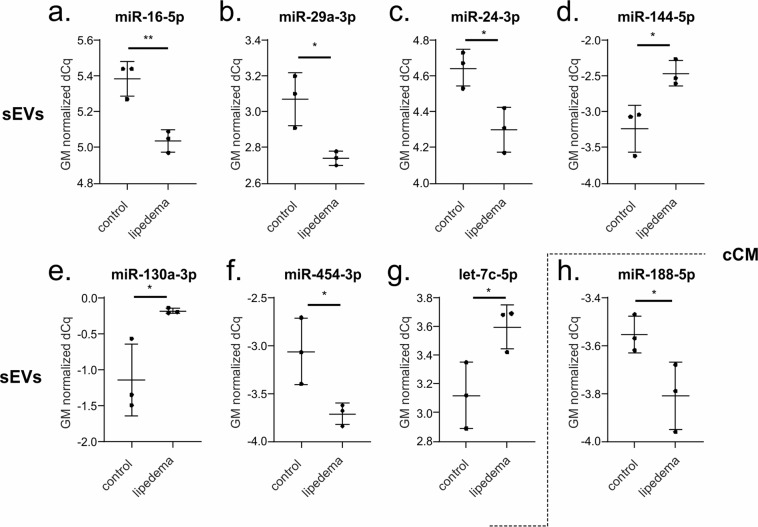
Scatterplots depicting global mean normalized levels of significantly regulated microRNAs (miRNAs) in small extracellular vesicles (sEVs) and concentrated conditioned medium (cCM) fraction.When applying a cut-off of p < 0.05, 7 miRNAs (3 up, 4 down) were identified to be significantly regulated in the sEV fraction: miR-16-5p (**a**), miR-29a-3p (**b**), miR-24-3p (**c**) and miR-454-3p **(f)** were downregulated in lipedema patients compared to controls, miR-144-5p (**d**), miR-130a-3p (**e**) and let-7c-5p (**g**) upregulated. One miRNA was identified to be significantly downregulated in the cCM fraction of lipedema patients compared to controls (**h**). n = 3 per group, unpaired two-tailed t-test, **p < 0.01, *p < 0.05.

The most variant miRNAs in the cCM and sEV enriched fraction were selected based on the coefficient of variation and used for hierarchical clustering (Fig. 3). While the variability in the cCM miRNA data was not primarily influenced by the disease (Fig. 3a, no clusters corresponding to the groups), this was the case for sEV enriched miRNA data, where 2 control samples clustered, and 2 lipedema samples clustered. Next, we performed statistical analysis to identify potentially regulated miRNAs. In general, the overall trend in up- and down-regulated miRNAs between lipedema and control was balanced for both the cCM and sEV enriched fraction (Fig. 4a,b). When applying a cut-off of p < 0.05, we observed one miRNA (miR-188-5p, p = 0.047) to be significantly down-regulated in the cCM fraction of lipedema patients compared to controls (Fig. 5h). Interestingly, this miRNA was not detected in the sEV fraction. Vice-versa, 7 miRNAs (3 up: miR–144-5p, miR-130a-3p, let-7c-5p; 4 down; miR–16-5p, miR-29a-3p, miR-24-3p, miR-454-p,) were identified to be significantly regulated in the sEV enriched fraction (Fig. 5a–g).

### Differentially regulated miRNAs analyzed for gene targets

In order to get insight into a biological potential impact of the differentially regulatedmiRNAs in lipedema, the 7 sEV- and 1 cCM- miRNAs were screened for experimentally validated miRNA/mRNA target pairs using miRNet. Pathway enrichment analysis was performed using the reactome pathway knowledgebase (Table 3). Thereby, we found in total 151 nodes comprising 144 target genes. To evaluate potentially impacted pathways, gene enrichment analysis of all annotated interactions between miRNAs and genes, identified 12 pathways with an adjusted p-value below 0.05. Among the pathways likely regulated by the 8 miRNAs were the NOTCH, Wnt and the SMAD/transforming growth factor beta (TGFβ) signaling pathway, and pathways involved in processes such as oxidative stress and senescence. miR-130a-3p and miR-454-3p were found to be the miRNAs with the largest interaction network comprising 122 and 123 different mRNAs, respectively (Supplementary Table 2).

**Table 3:** Reactome pathways identified by pathway enrichment analysis (www.mirnet.ca) for the differentially expressed miRNAs in lipedema versus control.

Reactomepathway enrichment analysis	#Genes	p-value
Oxidative Stress Induced Senescence	7	0,0044
Oncogene Induced Senescence	5	0,0044
Pre-NOTCH Transcription and Translation	4	0,0064
Pre-NOTCH Expression and Processing	4	0,0102
MicroRNA (miRNA) biogenesis	4	0,0102
Signaling by NOTCH	6	0,0137
SMAD2/SMAD3:SMAD4 heterotrimer regulates transcription	4	0,0137
Signaling by Wnt	9	0,0228
Cellular Senescence	7	0,0228
Signaling by TGF-beta Receptor Complex	5	0,0383
Gene Expression	17	0,0410
Transcriptional activity of SMAD2/SMAD3:SMAD4 heterotrimer	4	0,0438

mRNA targets for miR–16-5p, miR-29a-3p, miR-24-3p, and miR-454-p, miR–144-5p, miR-130a-3p, let-7c-5p and miR-188-5p, which significantly discriminate lipedema versus control, were identified based on experimental data supporting interactions. Reactome pathway enrichment analysis was performed by miRNet. Among the pathways likely regulated by the 8 miRNAs were the NOTCH, Wnt and the SMAD/transforming growth factor beta (TGFβ) signaling pathway, and pathways involved in oxidative stress and senescence. ^#^Genes = number of detected genes, hypergeometric test p-value < 0.05 = significant.

## Discussion

Since lipedema does not respond to lifestyle changes nor is there a treatment option other than liposuction of the diseased adipose tissue, a biomarker that would allow for identification of this disease and the prescription of liposuction by healthcare providers would be desirable. To date, diagnosis is based on clinical anamnesis, analytical weighing measurement and imaging of tissue composition by ultrasound. Additionally, non-invasive imaging such as MRI based quantification has been investigated. Adipose and sodium content in lipedema patients was found elevated in subcutaneous fat and skin, which hence has been suggested as potential biomarker for lipedema^29^. Since the disease is usually triggered by hormonal changes in females, a genetic susceptibility has been suggested^30^ (e.g. Williams-Beuern syndrome)^31^, however so far, no single gene causing the disease has been identified. The molecular load of EVs reflects the (patho-) physiological status of its original cells and thus can serve as a diagnostic tool, where sampling via liquid biopsies brings immense benefit to the physician and patient^32^. Besides the cell type-specific proteins, lipids and different classes of nucleic acids, EV contained miRNAs were found to be potent regulators in biological processes and in various diseases^33,34^. Numerous studies have already shown that the EV composition in diabetes, obesity, atherosclerosis, neurodegenerative diseases^35^, different cancers^36–38^ and renal damage^39–41^ distinguishes health and disease, which recommends them as potential diagnostic biomarkers. In fact, recent clinical trials have identified EVs as biomarker of prostate cancer^42^, Parkinson’s disease^43^ and difficult-to-treat arterial hypertension^44^.

Since miRNAs are crucial regulators of gene expression and are known to be involved in the progression of human diseases, our present study focused on extracellular miRNA in lipedema. Previously we identified that SVF cells from diseased subcutaneous WAT are altered in terms of adipogenesis, cell content and cellular subtype composition^7^. Thus, we sought to analyze the miRNA secretory profile of this cell population in lipedema versus healthy subjects (control). Considering the recent discussion about the relevance of extra- and intravesicular miRNAs^45^, we differentially analyzed the total and EV-contained miRNAssecreted by SVF from lipedemapatients and healthy controls. Turchinovich *et al*. postulated that extracellular miRNAs are predominantly non-vesicular, potentially a byproduct of dead cells but associated mostly with proteins^22^. In our study, the majority of the characterized miRNAs were indeed located extra-vesicular. However, we found that predominantly the EV-contained miRNA profile allowed discriminating healthy from lipedema, which was not the case for the secreted miRNA in the cCM, consisting mainly of extra-vesicular miRNAs. Additionally, when analyzing the surface marker profile of the EV preparations no significant differences between healthy individuals and lipedemapatients could be found. This demonstrates the importance of detailed characterization and especially the relevance of the EV cargo when characterizing the cellular status of a diseased tissue. We identifiedin our sEV preparations a significant reduction in miR–16-5p, miR-29a-3p, miR-24-3p, and miR-454-p, whereas miR–144-5p, miR-130a-3p and let-7c-5pwere significantly increased in lipedema compared to healthy controls. In the cCM, we found a significant reduction of one single miRNA, miR-188-5pin lipedema. MiR-188-5p has been described as a marker forage-related switch from osteogenic to adipogenic differentiation^46^. Further, miR-188-5p may have the potential to regulate migration and support of vascularization by mesenchymal stem cells (MSC), as previously described for a murine choroidal neovascularization model^47^. Deregulation of miR-188-5p might hence contribute to the endothelial barrier dysfunction and lymphangiopathy, which has been described in lipedema^48^. This suggests also a potential involvement of the MSC populations of the SVF in the progression of lipedema, since they are participating in the formation and stabilization of vessels^49^. It has been demonstrated that the total number of circulating particles (incl. their miRNA content) can be altered in disease^34,50^. In our study, particle number and size of enriched sEVs, analyzed by NTA, showed no difference between healthy individuals and lipedema patients. The majority of single miRNAsshowed higher abundance in cCM than in sEVs and only 9 of the analyzed miRNAs showed >50% signal originating from sEVs. miR-142-3p (80% sEV signal), was recently shown by another group to be selectively packaged in sEVs by a panel of oral squamous cell carcinoma cell lines, which resulted in lower intra-cellular levels and promoted malignant changes in these cells via increase of TGFBR1 expression^51^. Several miRNAs have recently been found to regulate adipose tissue biology and play a role in the development of obesity and related metabolic complications^52–55^. MiR-130 has been reported to inhibit adipocyte differentiation by the down-regulation of PPARγ^56,57^. Here, we found miR-130 significantly enhanced in lipedema which is in line with our previous work^7^ and the work of other groups^18^. Let-7 is one of the first miRNAs discovered. Knockout of let-7 family improved glucose tolerance in mice with diet-induced obesity^58^. In the present study, we observed a significant enhancement in let-7c-5p contents in sEVs from lipedema patients compared to healthy controls. Although we found significant increase in let-7c-5p, there is a consensus in the research field that lipedema does not favor the onset of diabetes, quite the opposite, lipedema negatively correlates with the development of insulin resistance. This is in line with observations by Pinnick *et al*. who found at cellular and miRNA level intrinsic differences between abdominal and gluteal adipose tissue. This result in an opposing metabolic disease risk, which is reduced forthe gluteal region – the site affected in lipedema^59,60^. Let-7c has been associated with regulation of macrophage polarization^61^. In a murine model of traumatic brain injury let-7c-5p protected mice from neuroinflammation and attenuated activation of microglia/macrophages^62^. This reported impact on macrophage polarization is also in consistence with another study demonstrating that miR-9, miR-127, miR-155, and miR-125b induce M1 polarization, while miR-124, miR-223, miR-34a, let-7c, miR-132, miR-146a, and miR-125a-5p promote M2 polarization in macrophages^63^. Since lipedema is an inflammatory disease accompanied by an infiltration of macrophages forming crown-like structure around adipocytes^9^, the local interplay of macrophages and cells of the diseased subcutaneous WAT, especially the contribution of EV miRNA communication, requires in depth investigation. When identifying potential target genes of the significantly up- and down-regulated miRNAs, we found regulatory molecules in Wnt signaling pathway, which play an important role in regulation of inflammation, oxidative stress and senescence^64^. Interestingly, our distinct secreted miRNAs were predicted to regulate a dynamic crosstalk of Wnt and another prominent pathway, NOTCH via targeting SMADs, which are also well-know nmediators of inflammatory, fibrotic, oxidative, proliferative, metabolic and angiogenic events^65–67^. The NOTCH super-signaling pathway plays an emerging role controlling key steps in the immune system and tissue homeostasis during repair and regeneration^68^. Notch interacts with SMADs to regulate blood vessel branching^69^ and promotes vascular sprouting through bone morphogenetic protein (BMP)/TGF-ß signaling^70^. The processes affected by regulation of the differentially regulated miRNAs - from oxidative stress to inflammation, angiogenesis, fibrosis have all been identified as part of the lipedema disease in the affected tissues^6,8,18,71^. This confirms the potential relevance of the identified SVF-secreted miRNAs in the cellular crosstalk during lipedema disease progression. As with the majority of studies, the design of the current study is subject to limitations. First, the sample size is too low to be able to control false-negative and false-positive rates sufficiently to claim the identified miRNAs more than biomarker candidates identified in a first discovery study. Second, when identifying sEV as the major source of the differentially regulated miRNA, we cannot exclude that even after the selected enrichment and purification steps, particles of other identity are enriched as stated in the latest misEV guidelines^72^. These data will now be useful to calculate power analysis for all future studies as it has become clear that differences exist. The important finding in this study is that differences in miRNA profile are mainly found in the sEV fraction. This indicates that in this disease, concerning SVF contribution, EVs rather than total RNA should be analyzed.

Here, we analyzed for the first time lipedema on miRNA level. Interestingly, all significantly regulated miRNAs found in this study may impact cellular processes that are reportedly affected by lipedema, such as adipogenesis, angiogenesis, inflammation and fat metabolism. We found a panel of 7 miRNAs that are influenced by lipedema in sEVs, whereas this was the case for only one miRNA in the total miRNA fraction. This study contributes to identify the role of SVF in the complex interplay of tissue components in lipedema on a miRNA level, where sEVs derived miRNAs were identified as the most relevant fraction.

## Supplementary information

Supplementary Information.
