# Supplementary material for: SVF-derived extracellular vesicles carry characteristic miRNAs in lipedema
Source: Sci Rep. 2020 Apr 29;10:7211. doi: 10.1038/s41598-020-64215-w (PMC7190633; doi:10.1038/s41598-020-64215-w)
Supplement: Supplementary file 1 — Supplementary Information. [file 41598_2020_64215_MOESM1_ESM.pdf]

SVF-derived extracellular vesicles carry characteristic miRNAs in lipedema

Eleni Priglinger[1,2*], Karin Strohmeier[1,2,4], Moritz Weigl[2,3], Carolin Lindner[1,2], Daniela Auer[2,5,6], Mario Gimona[2,5,6], Martin Barsch[7], Jaroslaw Jacak[2,4], Heinz Redl[1,2], Johannes Grillari[1,2,8], Matthias Sandhofer[7], Matthias Hackl[2,3#], Susanne Wolbank[1,2#]

[#]These authors contributed equally

[1]Ludwig Boltzmann Institute for Experimental and Clinical Traumatology, AUVA Research Center, Linz/Vienna, Austria
[2]Austrian Cluster for Tissue Regeneration, Vienna, Austria
[3]TAmiRNA GmbH, Vienna, Austria
[4]School of Medical Engineering and Applied Social Science, University of Applied Sciences Upper Austria, Linz, Austria
[5]Spinal Cord Injury and Tissue Regeneration Center Salzburg (SCI-TReCS), Salzburg, Austria
[6]GMP Laboratory, Paracelsus Medical University, Salzburg, Austria
[7]Austrian Center for Lipedema, Linz/Vienna, Austria
[8]Institute of Molecular Biotechnology, Department of Biotechnology, BOKU - University of Natural Resources and Life Sciences

***Corresponding Author:**
Dr. Eleni Priglinger
Eleni.Priglinger@trauma.lbg.ac.at
Ludwig Boltzmann Institute for Experimental and Clinical Traumatology
in the AUVA trauma research center, Austrian Cluster for Tissue Regeneration.
Donaueschingenstrasse 13
A-1200 Vienna, Austria

Supplementary Figure S1

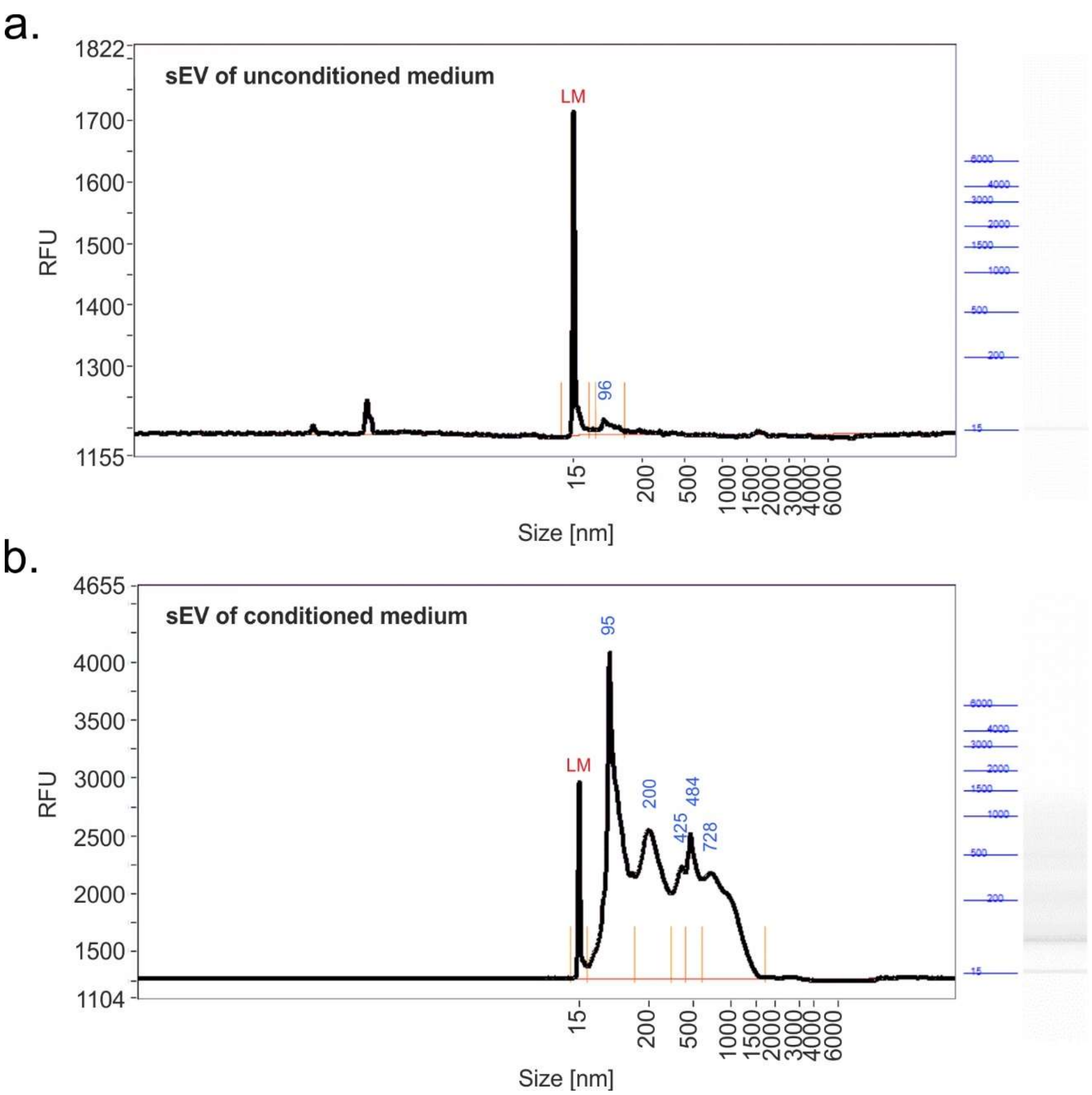

RNA concentration in unconditioned medium and cell conditioned medium. Assessment of RNA concentration via nanodrop analysis of total RNA concentration and subsequent analysis with the HS RNA Kit in small extracellular vesicle (sEV) fraction of unconditioned medium (a) and conditioned medium after 24 h of secretion (b), isolated via ultracentrifugation respectively. Nucleotide lengths of respective peaks are indicated as well as the signal derived from the lower marker (LM). RNA concentration in raw secretion medium (0.09 ng/µl) was negligible in comparison to RNA concentration observed in cell conditioned medium after 24 h of secretion (6.73 ng/µl).

Supplementary Figure S2

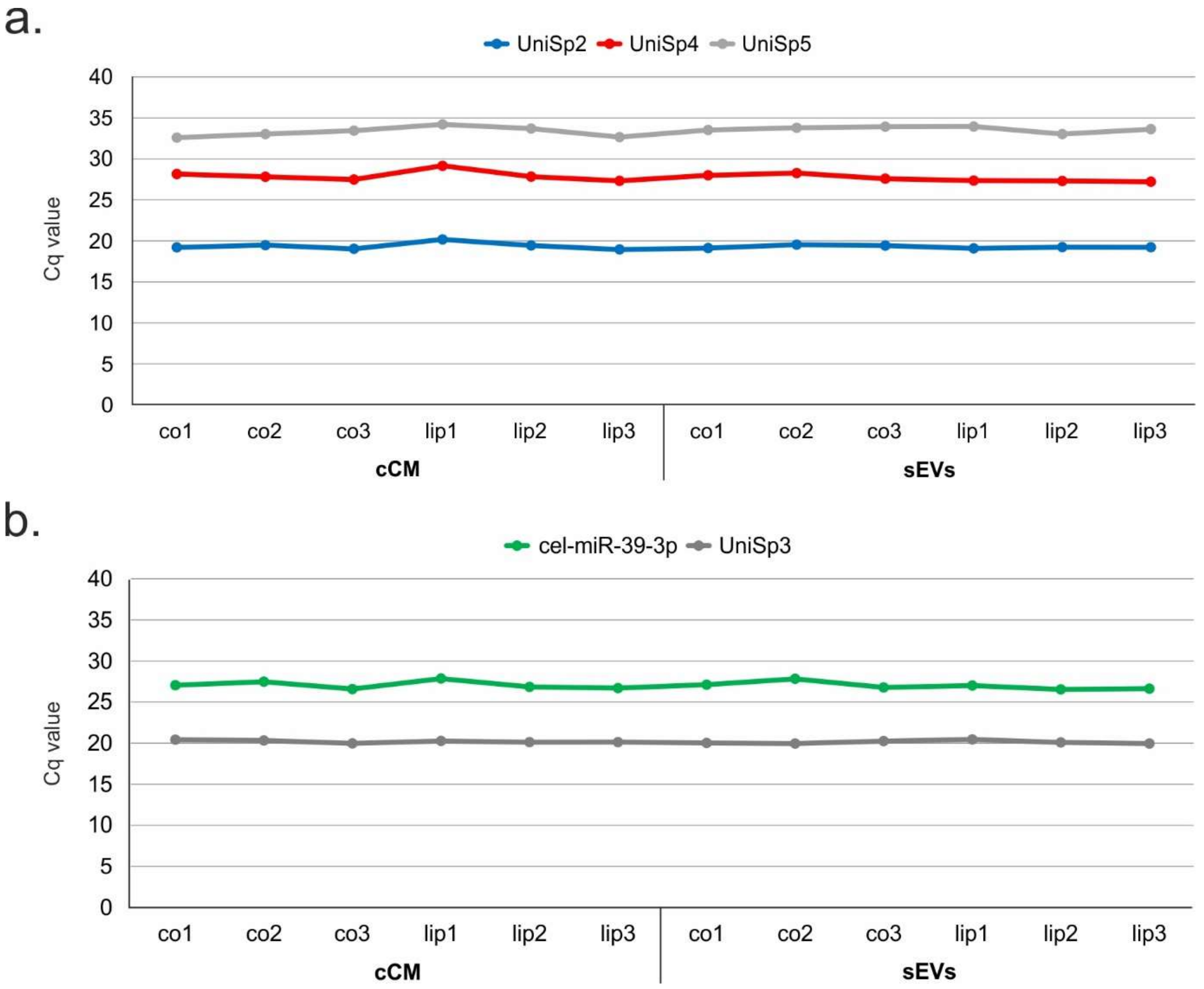

Quality control of RT-qPCR data. Data quality for all concentrated conditioned medium (cCM) and small extracellular vesicles (sEVs) samples from control (co) and lipedema (lip) was assessed using spike-in controls to determine RNA extraction efficiency, enzymatic inhibition, and overall variability. a) Cq-values for three distinct RNA spike-in controls with 100x (UniSp2), 1x (UniSp4), and 0.01x (UniSp5) concentration are shown. b) Cq-values for the spike-ins added during cDNA synthesis (cel-miR-39) and PCR amplification (UniSp3) are shown. All spike-in controls (a,b) showed homogenous values across all 12 samples with low variability.

Supplementary Figure S3

a.

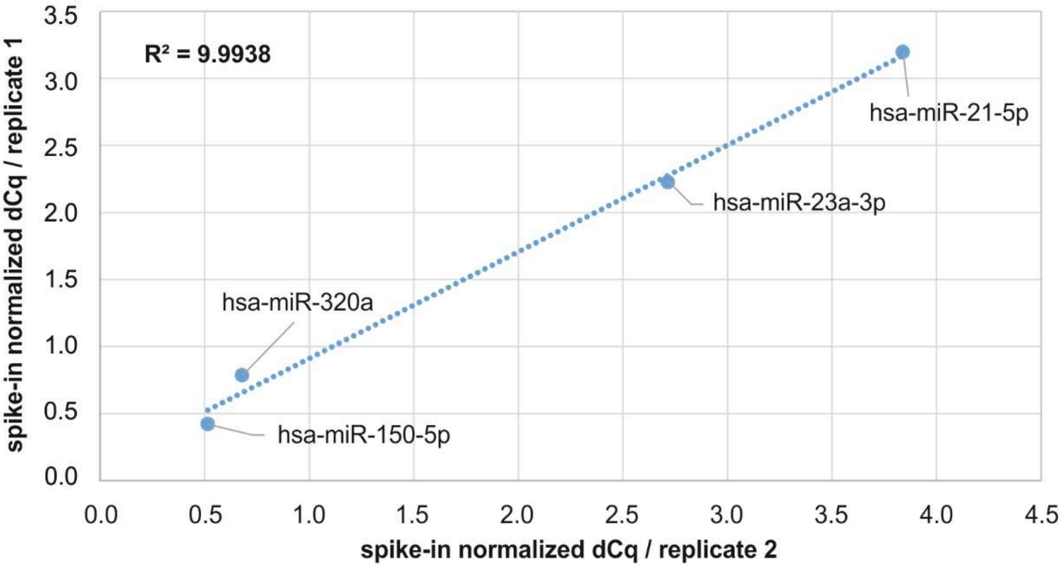

b.

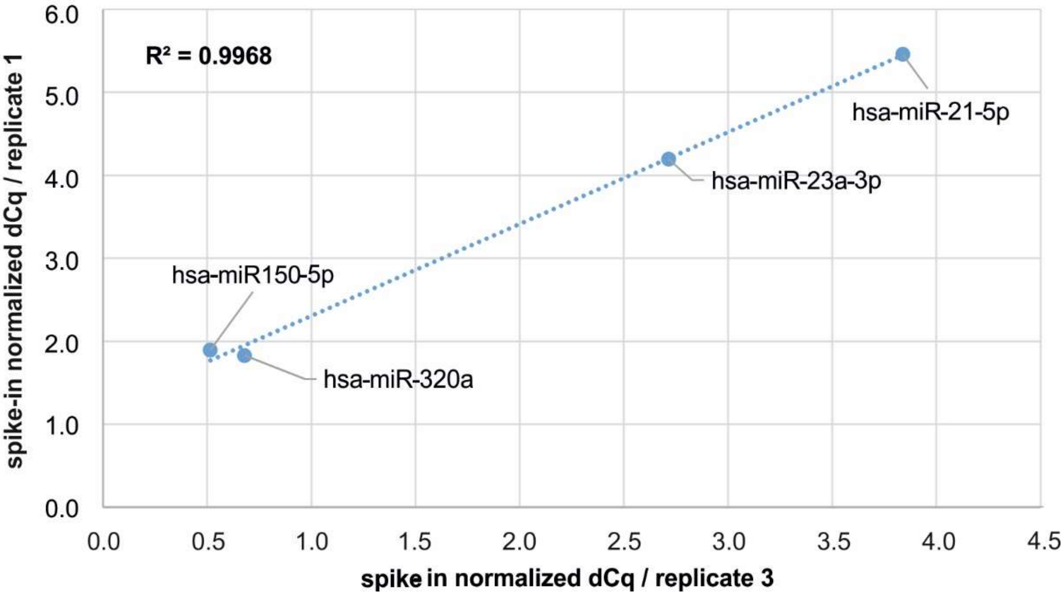

Technical reproducibility of conditioned medium samples derived from the same cell donor. Small extracellular vesicles enriched from three technical replicates of cell conditioned medium (24 h secretion) from one donor were analyzed for four microRNAs (miRNA) (miR-21-5p, miR-23a-3p, miR-320a, miR-150-5p) to assess the technical variability of the cell culture experiment. a) Spike-in normalized dCq values for replicate 1 (y-axis) vs. replicate 2 (x-axis). b) Spike-in normalized dCq values for

replicate 1 (y-axis) vs. replicate 3 (x-axis). High reproducibility of miRNA levels between technical replicates after 24 h of secretion was observed.

Supplementary Figure S4

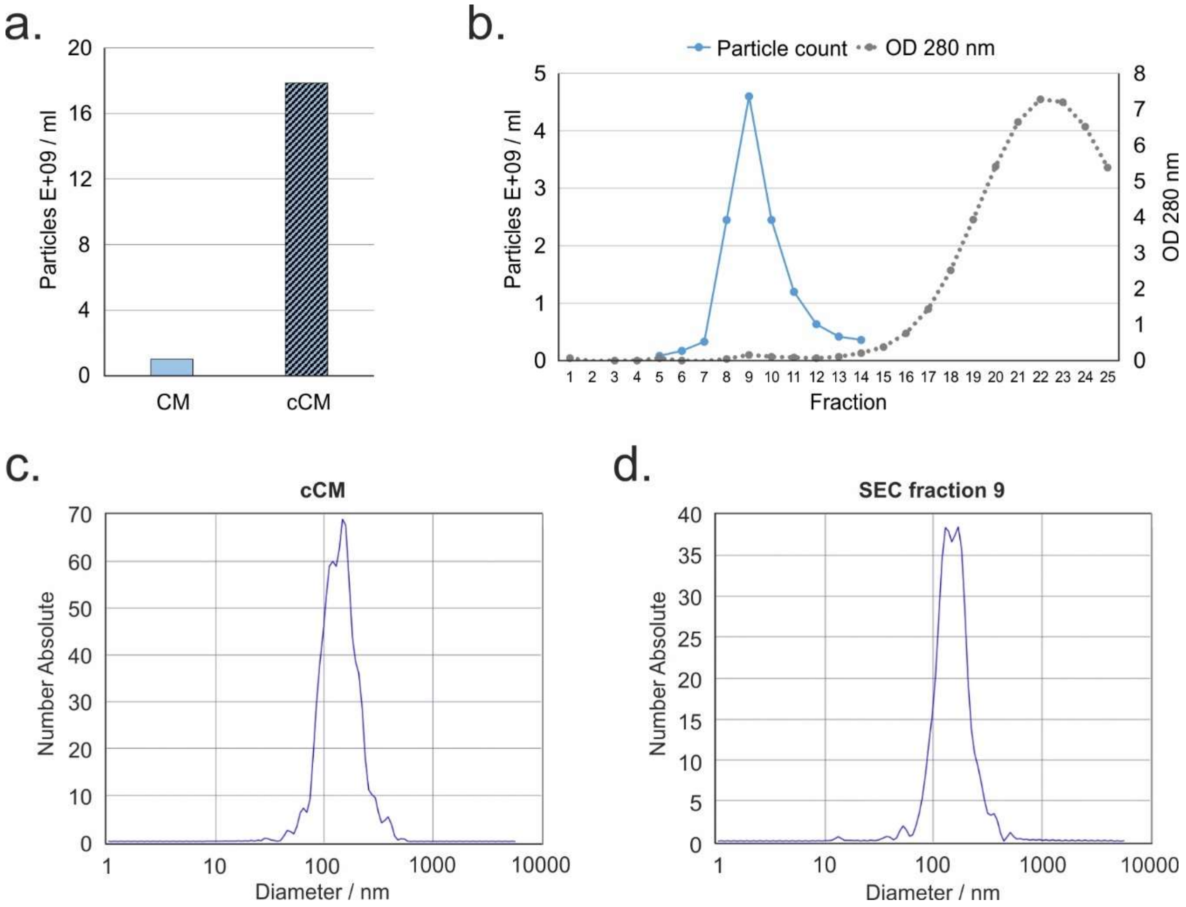

Nanoparticle tracking analysis of conditioned medium (CM), concentrated CM (cCM) and small extracellular vesicles (sEVs). a) Particle count of CM and cCM. b) cCM was fractionated by size exclusion chromatography. Particle count and protein measurement, obtained by absorbance at 280 nm, of obtained fractions. c) Size distribution of cCM and d) sEVs (exemplarily shown by fraction 9).

Supplementary Figure S5

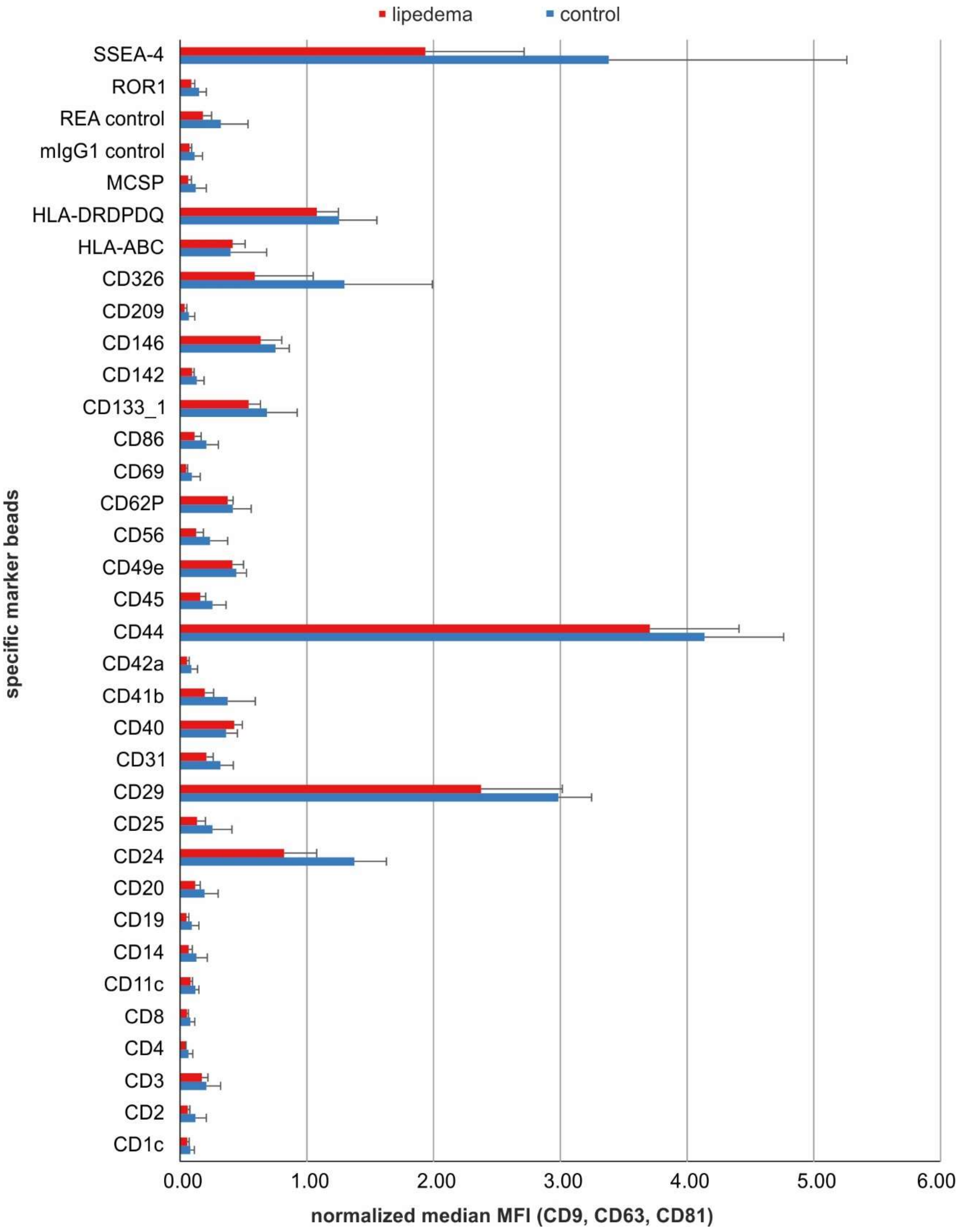

Surface marker analysis of small extracellular vesicles (sEVs) from healthy individuals and lipedema patients analyzed by multiplex bead-based flow cytometry assay. EV samples from healthy individuals (control) and lipedema patients

showed no significant differences in the surface marker profile. MFI=median fluorescence intensity. N=3.

Supplementary Table 1

No.	miRNA of EVs	Signal	No.	miRNA of EVs	Signal
1	hsa-miR-144-3p	122%	68	hsa-miR-93-5p	8%
2	hsa-miR-144-5p	92%	69	hsa-miR-152-3p	8%
3	hsa-miR-190a-5p	91%	70	hsa-miR-324-5p	8%
4	hsa-miR-142-3p	80%	71	hsa-miR-423-3p	7%
5	hsa-miR-454-3p	69%	72	hsa-miR-155-5p	7%
6	hsa-let-7a-5p	64%	73	hsa-miR-324-3p	7%
7	hsa-let-7d-5p	55%	74	hsa-miR-150-5p	7%
8	hsa-miR-331-3p	54%	75	hsa-miR-191-5p	7%
9	hsa-miR-103a-3p	53%	76	hsa-miR-484	7%
10	hsa-miR-107	47%	77	hsa-miR-140-3p	7%
11	hsa-let-7f-5p	42%	78	hsa-miR-154-5p	7%
12	hsa-miR-486-5p	37%	79	hsa-miR-328-3p	6%
13	hsa-let-7c-5p	36%	80	hsa-miR-195-5p	6%
14	hsa-miR-339-5p	32%	81	hsa-miR-223-3p	6%
15	hsa-let-7e-5p	32%	82	hsa-miR-221-3p	6%
16	hsa-miR-451a	28%	83	hsa-let-7d-3p	6%
17	hsa-miR-199b-5p	26%	84	hsa-miR-369-3p	6%
18	hsa-miR-100-5p	25%	85	hsa-let-7b-5p	6%
19	hsa-miR-99a-5p	25%	86	hsa-miR-29b-3p	6%
20	hsa-miR-31-5p	24%	87	hsa-miR-502-3p	6%
21	hsa-miR-22-3p	22%	88	hsa-miR-106a-5p	6%
22	hsa-miR-136-5p	20%	89	hsa-miR-24-3p	5%
23	hsa-miR-26a-5p	20%	90	hsa-miR-23b-3p	5%
24	hsa-miR-125b-5p	19%	91	hsa-miR-660-5p	5%
25	hsa-miR-196b-5p	19%	92	hsa-let-7b-3p	5%
26	hsa-miR-218-5p	19%	93	hsa-miR-30e-5p	5%
27	hsa-miR-98-5p	19%	94	hsa-miR-194-5p	5%
28	hsa-miR-301a-3p	19%	95	hsa-miR-320c	5%
29	hsa-miR-199a-5p	18%	96	hsa-miR-320b	5%
30	hsa-miR-127-3p	18%	97	hsa-miR-222-3p	5%
31	hsa-miR-590-3p	17%	98	hsa-miR-320a	5%
32	hsa-miR-148a-3p	16%	99	hsa-miR-20a-5p	5%
33	hsa-miR-26b-5p	15%	100	hsa-miR-21-5p	5%
34	hsa-miR-365a-3p	15%	101	hsa-miR-101-3p	5%
35	hsa-miR-18a-5p	14%	102	hsa-miR-185-5p	4%
36	hsa-miR-146b-5p	14%	103	hsa-miR-30e-3p	4%
37	hsa-miR-214-3p	14%	104	hsa-miR-23a-3p	4%
38	hsa-miR-140-5p	13%	105	hsa-miR-25-3p	4%
39	hsa-miR-30b-5p	12%	106	hsa-miR-361-5p	4%
40	hsa-miR-15b-5p	12%	107	hsa-miR-122-5p	4%
41	hsa-miR-374a-5p	12%	108	hsa-miR-10b-5p	4%
42	hsa-let-7g-5p	12%	109	hsa-miR-27a-3p	4%
43	hsa-miR-106b-5p	12%	110	hsa-miR-192-5p	4%
44	hsa-miR-30c-5p	11%	111	hsa-miR-425-3p	4%
45	hsa-miR-125a-5p	11%	112	hsa-miR-19b-3p	4%
46	hsa-miR-99b-5p	11%	113	hsa-miR-16-5p	4%
47	hsa-miR-18b-5p	11%	114	hsa-miR-186-5p	4%
48	hsa-miR-34a-5p	10%	115	hsa-miR-320d	4%
49	hsa-miR-15a-5p	10%	116	hsa-miR-19a-3p	4%
50	hsa-miR-92a-3p	10%	117	hsa-miR-130a-3p	4%
51	hsa-miR-382-5p	10%	118	hsa-miR-421	4%
52	hsa-miR-652-3p	10%	119	hsa-miR-342-3p	3%
53	hsa-miR-29a-3p	10%	120	hsa-miR-145-5p	3%
54	hsa-miR-425-5p	10%	121	hsa-miR-132-3p	3%
55	hsa-miR-376c-3p	10%	122	hsa-miR-215-5p	3%
56	hsa-miR-29c-3p	10%	123	hsa-miR-532-5p	3%
57	hsa-miR-126-3p	9%	124	hsa-miR-27b-3p	3%
58	hsa-miR-376a-3p	9%	125	hsa-miR-181a-5p	3%
59	hsa-miR-497-5p	9%	126	hsa-miR-590-5p	3%
60	hsa-miR-17-5p	9%	127	hsa-miR-146a-5p	3%
61	hsa-miR-374b-5p	9%	128	hsa-miR-145-3p	3%
62	hsa-miR-423-5p	9%	129	hsa-miR-143-3p	2%
63	hsa-miR-532-3p	9%	130	hsa-miR-30d-5p	2%
64	hsa-miR-424-5p	8%	131	hsa-miR-30a-5p	2%
65	hsa-miR-874-3p	8%	132	hsa-miR-378a-3p	2%
66	hsa-miR-193b-3p	8%	133	hsa-miR-181b-5p	2%
67	hsa-let-7i-5p	8%			

Analysis of microRNA (miRNA) signal origin from miRNAs in small extracellular vesicles (EVs). miRNAs signals obtained from all 133 miRNAs insEVs were compared against the total miRNA signal in concentrated conditioned medium (cCM) and expressed as %. For three miRNAs more than 90% of the signal in cCM originated from the sEV fraction, and for 6 further miRNAs >50% of the signal was derived from the sEV fraction. For 83 out of 133 miRNAs <10% of the total signal was obtained from the sEV fraction.

Supplementary Table 2

GeneID	hsa-let-7c-5p	hsa-mir-130a-3p	hsa-mir-144-5p	hsa-mir-16-5p	hsa-mir-188-5p	hsa-mir-24-3p	hsa-mir-29a-3p	hsa-mir-454-3p	Sum
DICER1	1	1		1			1	1	5
JARID2	1	1		1		1		1	5
MYC	1	1		1		1	1		5
NARS		1		1	1	1		1	5
TAOK1		1	1	1		1		1	5
ATP6V0E1		1		1		1		1	4
CCNA2		1				1	1	1	4
CCND1	1			1		1	1		4
CCND2		1		1			1	1	4
CDK4		1				1	1	1	4
DDX6		1		1			1	1	4
EREG		1				1	1	1	4
FAM217B		1	1			1		1	4
IGF2R		1		1		1		1	4
MAP3K9		1			1	1		1	4
MSANTD4		1		1		1		1	4
PAFAH1B2		1		1		1		1	4
POLR2D	1	1				1		1	4
PPP6R3		1		1	1			1	4
QKI	1	1					1	1	4
RAB11FIP1		1				1	1	1	4
RAN		1		1			1	1	4
RPRD2		1		1		1		1	4
SLC35E2B		1		1		1		1	4
TSC22D2	1			1	1	1			4
ACBD5		1		1				1	3
ACP6		1				1		1	3
ACVR1		2						1	3
ADARB2		1	1					1	3
AGO3		1				1		1	3
ANKRD9		1				1		1	3
ARHGAP12		1		1				1	3
ARL17B		1				1		1	3
ARL6IP1	1	1						1	3
ATG9A	1			1			1		3
ATP6V1B2		1		1				1	3
BCL7A				1		1	1		3

BTBD3		1				1		1	3
BZW1	1			1		1			3
C11orf57	1	1						1	3
CBX6	1			1			1		3
CCNT2	1			1			1		3
CDADC1		1		1				1	3
CDCA4		1		1				1	3
CEP55		1		1				1	3
CFL2		1		1				1	3
CHERP		1		1				1	3
CHIC1		1		1				1	3
CINP		1				1		1	3
COX20		1				1		1	3
CSNK2A1	1	1						1	3
CUL3		1		1				1	3
CYP20A1		1				1		1	3
DCAF7	1			1		1			3
DEPDC1		1				1		1	3
DIABLO	1			1			1		3
DLC1		1		1				1	3
EDN1	1	1						1	3
ENPP4		1		1				1	3
FOXQ1		1				1		1	3
FZD6		1		1				1	3
GMFB		1				1		1	3
GPR82		1				1		1	3
GPRC5A		1		1				1	3
HBP1		1					1	1	3
HSPA8		1		1				1	3
IER3IP1		1		1				1	3
IFNLR1	1	1						1	3
KLHL15				1		1		1	3
KREMEN1	1	1						1	3
LEFTY1	1	1						1	3
MAP7		1		1				1	3
MAPKAPK5		1				1		1	3
MBNL1		1			1			1	3
MDM4	1		1			1			3
METAP2	1			1		1			3
MLEC		1				1		1	3
MREG		1	1					1	3
NCAPD2		1		1				1	3
NFE2L1		1				1		1	3
NIPA1		1				1		1	3
NOM1	1	1						1	3
NPTX1	1	1						1	3
PDLIM5	1			1		1			3
PDP2	1	1						1	3
PEX13		1		1				1	3
PIGA		1	1					1	3
PLAG1				1	1		1		3
PLEKHS1		1				1		1	3
POGZ		1				1		1	3
POLR1B		1				1		1	3
PPIG		1		1				1	3
PPM1D				1		1	1		3
PPP1R15B	1	1						1	3
PRKAA1		1		1				1	3
PRNP		1		1				1	3
PRRG4		1				1		1	3
PSMB5		1		1				1	3
RAB34	1	1						1	3
RAB5B	1	1						1	3
RACGAP1		1		1				1	3
RAP2C				1		1		1	3

REL				1	1		1		3
RFC2	1	1						1	3
RFT1		1		1				1	3
RNF11		1				1		1	3
RNF149		1		1				1	3
RPS6KA5		1				1		1	3
S1PR2		1				1		1	3
SALL3	1	1						1	3
SIK1		1		1				1	3
SLC10A3	1	1						1	3
SLC11A2	1			1		1			3
SLC12A7	1	1						1	3
SLC38A2		1		1				1	3
SMAD4		1	1					1	3
SMTNL2		1				1		1	3
SNTB1		1				1		1	3
SNTB2		1		1				1	3
SPATA5				1	1	1			3
STX16		1				1		1	3
TMTC1		1			1			1	3
TMTC3	1			1			1		3
TNFRSF10B	1	1						1	3
TNRC6B		1		1				1	3
TP53				1		1		1	3
TRIM4		1		1				1	3
TRIM71	1	1						1	3
TSPAN3		1		1				1	3
TUBB2A	1			1			1		3
TXNIP		1		1				1	3
UBC		1				1		1	3
UBN2		1		1				1	3
WASL	1	1						1	3
WNK3		1		1				1	3
XIAP		1				1		1	3
ZBTB5	1			1			1		3
ZFYVE9		1				1		1	3
ZMAT3		1		1				1	3
ZNF107		1				1		1	3
ZNF317		1				1		1	3
ZNF417	1	1						1	3
ZNF529		1	1					1	3
ZNF620		1		1				1	3

List of genes that were found by mirnet to interact with at least 2 of the differentially regulated microRNAs.
